# New Jersey COVID-19 municipal dataset

**DOI:** 10.1016/j.dib.2021.107426

**Published:** 2021-09-24

**Authors:** Yuqi Wang, Sarah R. Allred, Emily A. Greenfield, Aayush Yadav, Ryan Pletcher, George Arthur, Sachin Saxena, Trista Harig, Emily Rankin, Benjamin Rudolph, Ummulkhayer Sameha, Shwetal Sharma, Shibin Yan

**Affiliations:** a School of Social Work, Rutgers University, New Brunswick, USA; b Senator Walter Rand Institute for Public Affairs, Rutgers University, Camden, USA; c Department of Psychology, Rutgers University, Camden, USA; d Department of Computer Science, Rutgers University, Camden, USA; e Department of Mathematics, Rutgers University, Camden, USA; f Department of Public Affairs, Rutgers University, Camden, USA

**Keywords:** SARS-CoV-2, Case rate, Pandemic, Public health, Geospatial analysis

## Abstract

Although data about COVID-19 cases and deaths in the United States are readily available at the county-level, datasets on smaller geographic areas are limited. County-level data have been used to identify geospatial patterns of COVID-19 spread and, in conjunction with sociodemographic variables, have helped identify population health disparities concerning COVID-19 in the US. Municipality-level data are essential for advancing more targeted and nuanced understanding of geographic-based risk and resilience associated with COVID-19. We created a dataset that tracks COVID-19 cases and deaths by municipalities in the state of New Jersey (NJ), US, from April 22, 2020 to December 31, 2020. Data were drawn primarily from official county and municipality websites. The dataset is a spreadsheet containing cumulative case counts and case rates in each municipaly on three target dates, representing the peak of the first wave, the summer trough after the first wave, and the outbreak of the second wave in NJ. This dataset is valuable for four main reasons. First, the dataset is unique, because New Jersey's Health Department does not release COVID-19 data for the 77% (433/565) of municipalities with populations smaller than 20,000 individuals. Second, especially when combined with other data sources, such as publicly available sociodemographic data, this dataset can be used to advance epidemiological research on geographic differences in COVID-19, as well as to inform decision-making concerning the allocation of resources in response to the pandemic (*e.g.*, strategies for targeted vaccine outreach campaigns). Third, county-level data mask important variations across municipalities, so municipality-level data permit a more nuanced exploration of health disparities related to local demographics, socioeconomic conditions, and access to resources and services. New Jersey is a good state to explore these patterns, because it is the most densely-populated and racially/ethnically diverse state in the US. Fourth, New Jersey was one of the few locations in the US with a high prevalence of COVID-19 during the first wave of the pandemic in the US. Thus, this dataset permits exploration of whether sociodemographic variables predicted COVID-19 differently as time progressed. To summarize, this unique municipality-level dataset in a diverse state with high COVID-19 cases is valuable for scholars and policy analysts to explore social and environmental factors related to the prevalence and transmission of COVID-19 in the US.

## Specifications Table

**Table:** 

Subject	Infectious Diseases
Specific subject area	COVID-19 cases and deaths at municipal-level, State-wide data in US, spatiotemporal dataset for exploring trends and distributions of COVID-19
Type of data	Table
How data were acquired	The dataset was created by compiling municipal-level data from public announcements by local officials across New Jersey (NJ) on COVID-19 cases from April 22, 2020 to December 31, 2020. We created a line graph to visualize the trend of changes across the state. Three target dates were selected to represent the spring peak of the first wave of COVID-19 (April 27, 2020), the summer trough after the first wave (June 30, 2020), and the outbreak of the second wave (December 13, 2020). The cumulative case counts and rates were calculated for the three target dates for each municipality. Because not every municipality has valid case counts for each day, we selected the nearest date from the target dates for calculating the cumulative cases.
Data format	Raw Filtered
Parameters for data collection	Data in public announcements by local officials on cases and case rates reported at the municipal-level across NJ.
Description of data collection	A team of students and staff recorded numbers of reports of COVID-19 cases at the municipal-level across New Jersey every one to three days since April 22, 2020, to December 31, 2020. Primary data sources included county health department websites and municipal websites. A full list of data sources can be found in the supplemental material.
	From the raw data, we plotted a line graph to select three dates that represent key time points during the pandemic in NJ. We reported the cumulative case counts and rates on the three target dates. If there were missing data on the target dates, an algorithm selected the closest date with valid data within an 11-day window centered on the target date. To detect errors, an algorithm in MATLAB identified outliers for the cumulative case rates. We then manually checked the sources of error for each identified outlier.
Data source location	There are a total of 565 municipalities in New Jersey, US. A total of 560 municipalities reported valid cumulated case counts on or around April 27, 2020. A total of 551 municipalities reported valid cumulated case counts on or around June 30, 2020. A total of 510 municipalities reported valid cumulated case counts on or around December 13, 2020. Users can refer to the dataset file for the full list of municipalities and counties with valid data (variable names: Municipality and County).
Data accessibility	Data can be accessed through the link: http://dx.doi.org/10.17632/c6yg37mhb4.2 The uncleaned dataset with raw cases and deaths per day by municipality can be requested by emailing the author(s) at srallred@camden.rutgers.edu.

## Value of the Data

• As the COVID-19 pandemic has unfolded across the globe, studies on the prevalence of COVID-19 have demonstrated how people's risk of becoming infected depends, in part, on their geospatial locations. While county-level COVID-19 data are readily available, data at smaller geographic units are less available in the United States. Most US counties contain many municipalities with differing levels of socioeconomic composition and geographic characteristics. Thus, aggregating municipality data to the county level can mask important variations across municipalities.
• The dataset can be used by researchers to examine the spatiotemporal patterns of COVID-19 as well as to explore risk and protective factors by geographic communities in NJ. Public health and related authorities at the state, county, and local levels can use the data to guide policy-making and resource allocation. Community residents and leaders can use the cumulative rates and maps to easily assess the severity of COVID-19 in their communities and to guide practices.
• Geographic variations in case rates have been used across the globe for immediate, practical purposes, such as when to shut down schools and businesses and to help guide individual choices about travel and recreation. Geographic variations in case rates can also be used for more conceptual purposes, such as to understand ways that sociodemographic characteristics at the community level, such as income and racial composition, might predict risk for COVID-19 infection.
• New Jersey is a valuable setting to examine spatiotemporal patterns of COVID-19 because it is one of the few areas with high case rates during the first wave of the pandemic and because of its diversity in terms of race/ethnicity, socioeconomic status, and land use. This diversity suggests that patterns across the state may be generalized to accelerate analyses at the national and cross-national levels.
• Inaccuracies in reporting and recording COVID-19 data are common. We identify errors and outliers, clean the data, and select three key dates for reporting and estimating the cumulative cases. The technical report describes in detail the methods used to assure data quality. The data are in a user-friendly spreadsheet with geographic identifiers that can be combined with census data and other geographic data.

## 1 Data Description

The dataset includes a spreadsheet with cumulative cases and case rates by municipality in NJ as recorded from local-level reports on or around three target dates. We present field names and field descriptions for the datafile in Table 1. In addition to municipality and county names, we also included the 10-digit Federal Information Processing System (FIPS) codes as the unique identifier for each municipality (county subdivision). Population data were extracted from the 2010 US Decennial Census Data [1], and all cumulative rates (cases per 100K) were calculated based on the 2010 population. The three target dates represent three main stages of COVID-19 spread in NJ: April 27, 2020, was around the peak of the first wave (spring peak); June 30, 2020, was when the curve of the first wave was flattened (summer trough); December 13, 2020, was around the outbreak of the second wave. For municipalities with missing data on the target dates, we selected the closest dates from target date the with valid data to calculate the cumulative cases. The actual date used are reported in ActualDateSpring, ActualDateSummer, and ActualDateFall. A total of 560 out of the 565 municipalities reported valid cumulative cases on or around April 27, 2020. The number dropped to 551 on or around June 30, 2020, and to 510 on or around December 13, 2020. A public-facing technical report describing the dataset has been released together with the dataset [2].

**Table 1:** A Summary of data fields.

Column#	Field name	Field Description
1	Municipality	Name of municipality
2	County	County where municipality is located
3	FIPS 2010	FIPS codes of the municipality
4	Population 2010	Population reported in the 2010 census
5	Total Cases On 27-Apr-2020	Cumulative *number* of positive COVID-19 tests reported in the municipality for the spring peak
6	Actual Date Spring	Indicates the actual date used for the spring peak if data were not recorded on April 27.
7	Cases Per 100 K Spring	Cumulative *rate* of positive COVID-19 tests per 100,000 population for the spring peak
8	Total Cases On 30-Jun-2020	Cumulative *number* of positive COVID-19 tests reported in the municipality for the summer trough
9	Actual Date Summer	Indicates the actual date used for the summer trough if data were not recorded on June 30
10	Cases Per 100 K Summer	Cumulative *rate* of positive COVID-19 tests per 100,000 population for the summer trough
11	Total Cases On 13-Dec-2020	Cumulative *number* of positive COVID-19 tests reported in the municipality for the late fall peak
12	Actual Date Fall	Indicates the actual date used for the late fall peak if data were not recorded on December 13
13	Cases Per 100 K Fall	Cumulative *rate* of positive COVID-19 tests per 100,000 population for the late fall peak

**Fig. 1:**
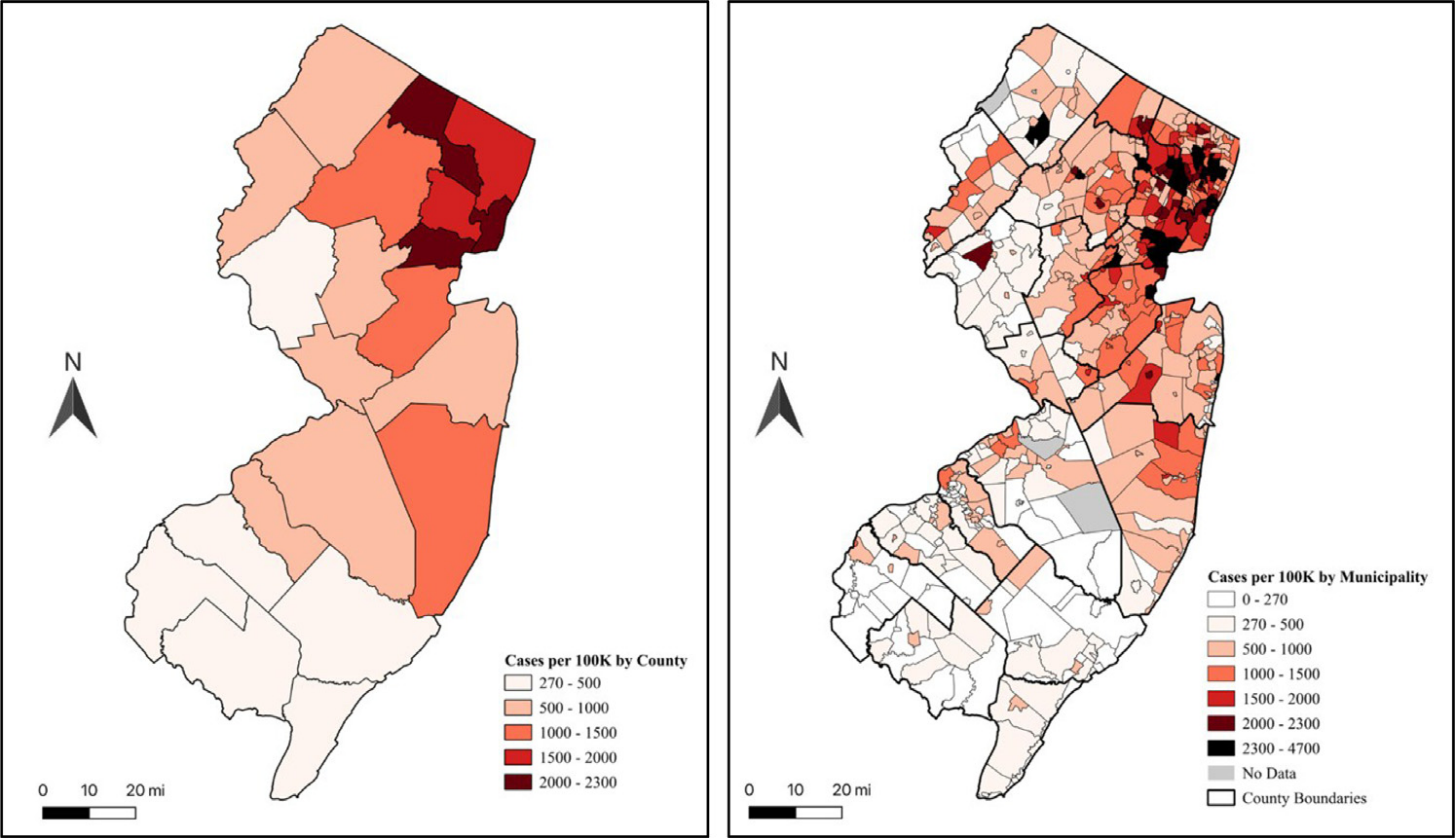
Cumulative cases per 100 K by county and municipality: April 27, 2020.

**Fig. 2:**
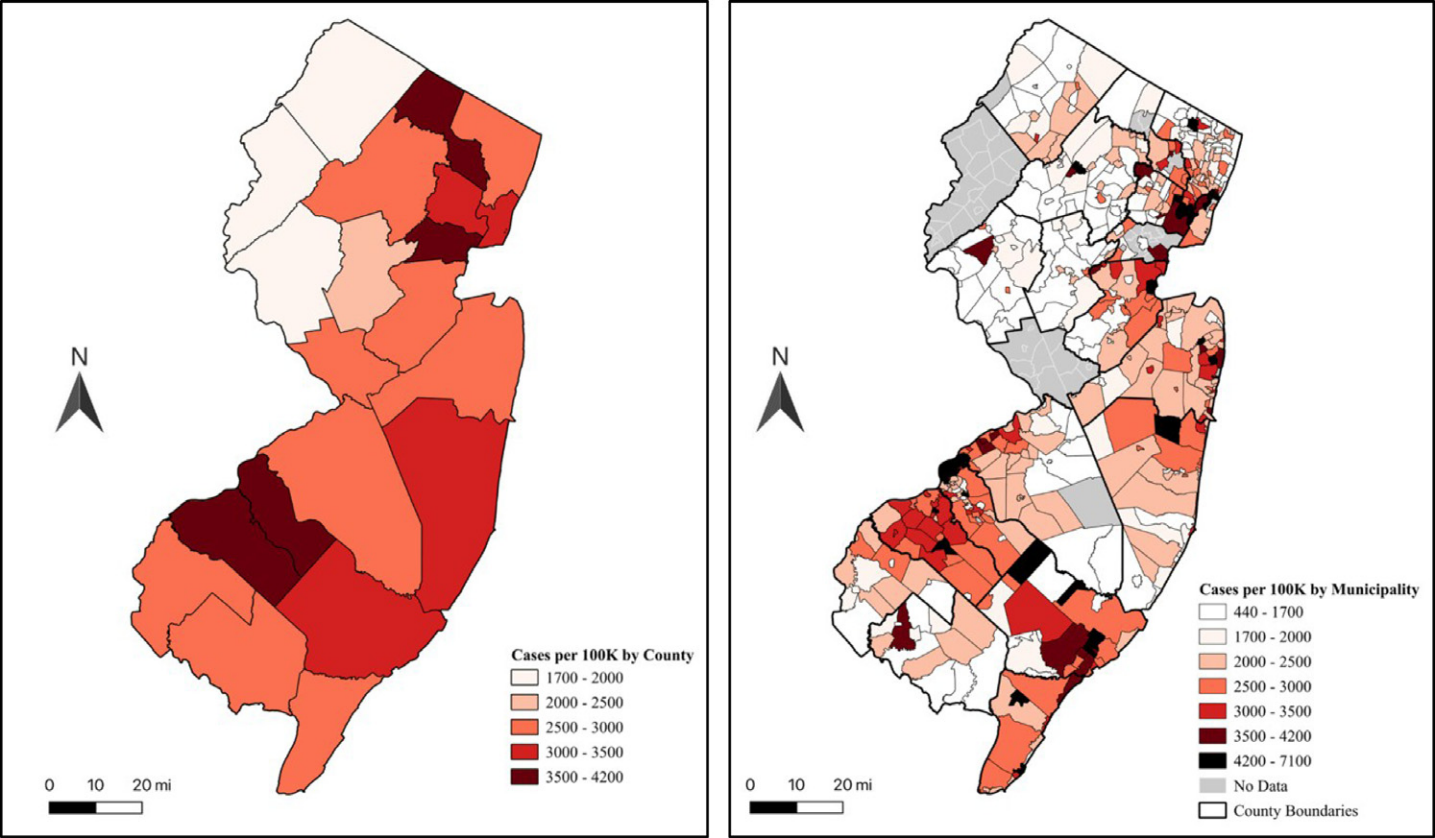
Cumulative cases per 100K by county and municipality: June 30, 2020 - December 13, 2020 (Second Wave).

To facilitate the usage of the dataset, we created maps for public use from our datafile. We include maps for both County-level data from Johns Hopkins Coronavirus (JHU) Database [3] and the NJ municipal-level dataset. The maps show the cumulative case rates per 100,000 residents in graduated colors. We set the cut-off values for the municipal map to match the corresponding county map to demonstrate how the municipal-level case rates vary from the county-level case rates. Fig. 1 visualizes cumulative COVID-19 case rates of counties in contrast to municipalities through April 27, 2020, representing the case rates since the start of COVID-19 until the peak of the first wave. Fig. 2 shows the case rates of counties in contrast to municipalities during the second wave, which is defined as the cumulative case rate between June 30, 2020, and December 13, 2020. Darker colors indicate higher prevalence of COVID-19.

## 2 Experimental Design, Materials and Methods

### 2.1 Collect raw COVID-19 data

A team of students and staff recorded the number of cumulative cases at the municipal level, as reported by local officials in NJ, from April 22, 2020, to December 31, 2020. We searched for the numbers reported in a list of official or other trustworthy sources, including county health departments and municipal Facebook pages. A completed list of data sources is reported in the supplemental materials. Students were instructed to record data in a spreadsheet every one to three days. However, there are some gaps when the data were not reported by any source. We collected and released data because the official COVID-19 data collected at the municipal level was not available to the public. Meanwhile, studies on COVID-19 spread have suggested using smaller geographic units to examine the spatiotemporal patterns of COVID-19 [4].

The goal of the data collection was to balance the time of manually recording many data points against the need for data over time. If every data point was reported on every day, the data set would include 143,510 data points (254 days × 565 municipalities). The final data set includes 80,741 data points (56%).

Through the data collection, we recognized the possible inaccuracies from the data sources, particularly at the early stage of the COVID-19 pandemic. There was a delay between cases collected by local health officials and cases submitted to the state-wide tracking system, the Communicable Disease Reporting and Surveillance System (CDRSS) [5]. There were also periodic changes in the local recording processes and criteria for a positive test result. Therefore, cumulative cases reported on a given day can be updated in a later day. Conflicting case counts also occur in the widely used JHU Database [3]. To provide some sense of the frequency of revision, we examined how often cumulative case counts decrease in the JHU Database. Since the number of cases cannot in fact decrease, the number of reported decreases provides some sense of how often revisions occur. Of the 5,355 data points in the JHU database for New Jersey between April 22, 2020 and December 31, 2020 (21 counties × 255 days), 73 (1.4%) show this sort of decrease in cumulative cases.

To check the occurrences of the conflicting cases of the municipal data in comparison to the JHU Dataset, we next calculated how often municipality case counts decreased on subsequent days, and how often county case counts, aggregated across the municipalities within the county, decreased. Because the county unit is an aggregate of municipalities, case decreases in some municipalities may be masked by case increases in other municipalities. Thus, there is an a priori expectation that there will be higher error rates at the municipality level than are detectable at the county level. At the municipality level, we found that 4,923 of 80,741 data points (6.1%) showed decreases, while county-level data aggregated from the municipality dataset showed decreases in 75 out of 2,738 data points (2.7%). Although the numbers cannot be directly compared to the JHU Dataset, mainly because the municipality dataset does not contain data for every day, the rates of conflicting cases of the municipality dataset are broadly consistent to the 1.4% rate of the JHU Dataset.

Although it is not plausible to verify the accuracy of each data entry, the data capture the overall trends over time for each municipality. In order to balance the risk of errors on individual days with the value of the municipality data over time, we selected three key target dates and reported the cumulative cases on the three dates. We adopted several data cleaning techniques to minimize missing data and errors in the final datafile, as discussed in the following sections.

### 2.2 Select the target dates

**Fig. 3:**
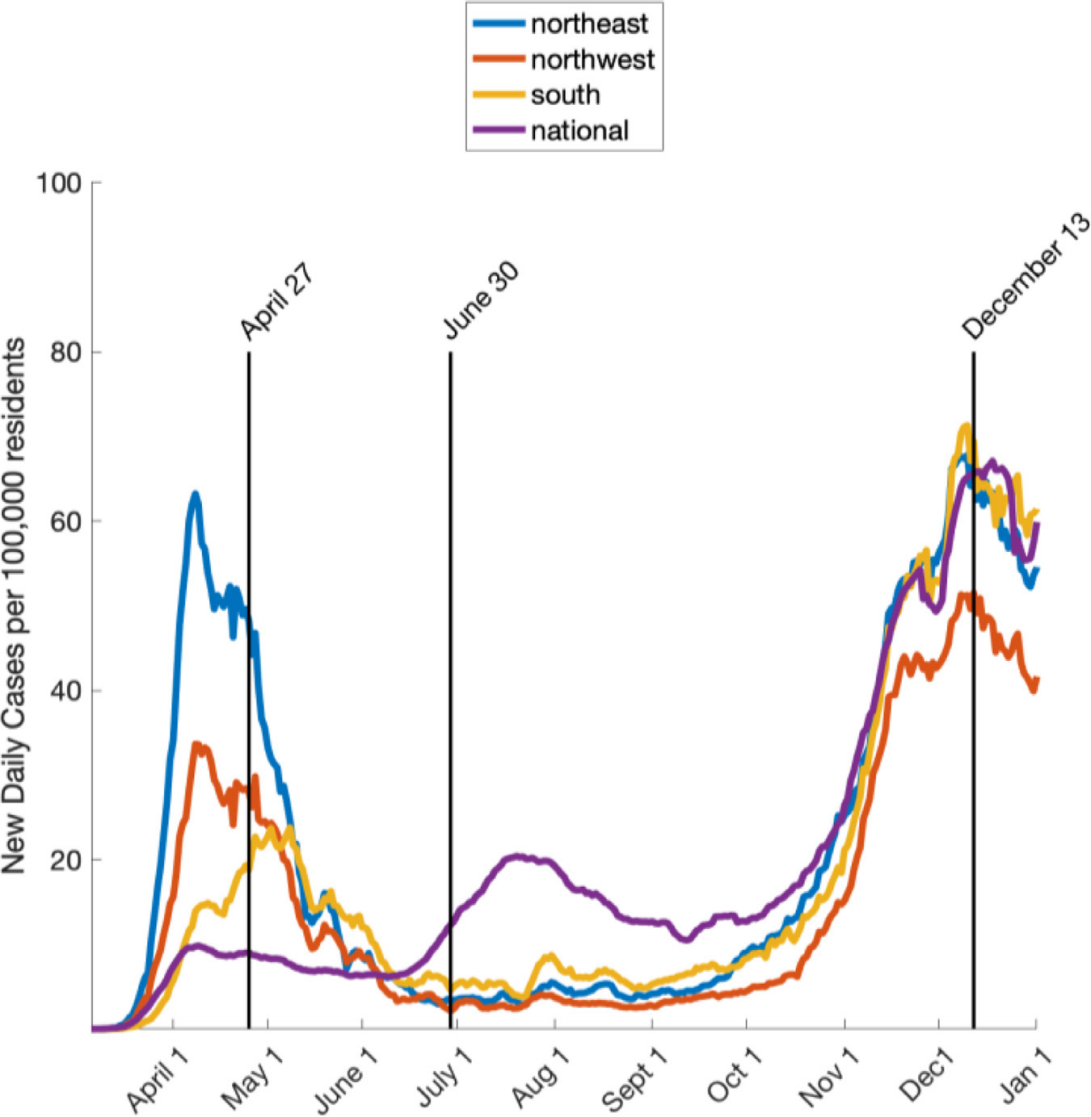
New daily cases per 100 K (January 22, 2020 – December 31, 2020) for the US and three NJ regions. *Notes:* Northeast New Jersey consisted of counties that had more than 1,000 COVID-19 cases on April 1, 2020t (Bergen, Essex, Hudson, Middlesex, Monmouth, Ocean); Northwest New Jersey consisted of counties that had between 250 and 1000 cases on April 1, 2020 (Hunterdon, Mercer, Morris, Somerset, Sussex and Warren); Southern New Jersey consisted of counties that had fewer than 250 cases on April 1, 2020 (Atlantic, Burlington, Camden, Cape May, Cumberland, Gloucester).

To select target dates that represent key time points of COVID-19 spread in NJ, we portrayed the case rates (per 100,000 residents) per day over time for three different regions across NJ in a line chart (see Fig. 3). The line chart depicts the trends of COVID-19 from January 22, 2020 (the first date of data in the Johns Hopkins data), to December 31st, 2020, in NJ. The first cases of COVID-19 in NJ were in its northeast counties, near the New York City epicenter of the first outbreak of COVID-19 in the US. COVID-19 cases were then identified in northwestern counties in New Jersey, and finally in southern regions. Thus, we distinguished these three regions in NJ (see Fig. 1) and compared the trends of COVID-19 in these three regions to the national trend from January 22, 2020, to December 31, 2020. The new cases reported per day per 100,000 residents (based on the population from 2010 census data) are shown in the y-axis. The plotted value for each day is the average of the case rate for the preceding seven days. The 7-day window is standard and accounts for weekly variations in reporting. The case rate on each day was calculated by summing the total number of cases within the region and dividing by the population of the region. The line graph compares broad regional trends over time and informed the target dates used for reporting the cumulative cases in the published datafile.

Based on the trends of changes of the three regions in NJ, the team discussed selecting the most informative cut-off dates for calculating the cumulative cases. The team reached a consensus on the three key dates. Although there was a delay in reaching the peak of the first wave for the Southern region, the date of April 27, 2020, generally represents the peak of the first wave in NJ. On June 30, all three regions reached the lowest points of new case rates, which represents the end of the first wave. The second wave started gradually, but roughly on December 13, 2020, when the new case rates reached the peak that followed by decreases.

Because not all municipalities reported valid cumulative cases for the target dates, we created a computer algorithm to select the data from a date closest to the target dates. The algorithm searched dates in the following order of distance from the target date [0, −1, 1, −2, 2, −3, 3, −4, 4, −5, 5]. For example, if the data on the target date of June 30 is missing, the algorithm checked the prior date of June 29, then July 1, then June 28, and so on. In Table 2, we summarized which date was used for the data. If no data were reported within the 11-day window for a given municipality, we treat it as missing data. The majority of the municipalities reported valid data. However, the count of valid data drops from 560 to 551 and to 510 over time. Similarly, availability of data on the target date also drops from 94 to 79% and then to only 48%. Also, we calculated “shift” as the absolute values of the differences in dates between the target date and the actual date. On April 27, 2020, the average variation of dates from the target date is 0.06, while on December 13, 2020, the average variation of dates from the target date is 0.75. This increase in “shift” indicates that as the pandemic progressed in 2020, fewer local officials continued to announce their data on COVID-19 cases, and/or officials released their municipal-level data less frequently.

**Table 2:** Target dates and the actual dates used.

	Total valid data	% on target date	Average ``shift''*
April 27, 2020	560	94%	0.06
June 30, 2020	551	79%	0.53
December 13, 2020	510	48%	0.75

⁎ “Shift” was calculated as the absolute values of the differences in dates between the target date and the actual date. Shifts were averaged across all municipalities to indicate the overall variability in dates recorded.

### 2.3 Flag errors in data

One potential source of inaccuracy is transcription errors. As cases reported in the data sources are not in a downloadable datafile, rather numbers reported on a website or dashboard, the team manually recorded the data into the spreadsheet, resulting in possible transcription errors. Errors that are common in transcription (such as transposing two digits from 19 to 91) could lead to a drastically incorrect interpretation.

We created a MATLAB program to identify such errors in the raw data for new daily case rates. Conceptually, the goal is to flag data points for inspection that seem unusually large or small and might indicate a transcription error. This is not trivial, because the nature of this data set (cumulative case counts) is to grow over time. Thus, the task of the algorithm is to determine whether a *particular* data point is outside what is expected by the *trend* of the data set as a whole. In such time-series analysis, the standard procedure is to stationarize the time-series (or colloquially, to remove the *trend* from the data; see, for example [6]). In order to accomplish this, we take the second-order difference of the cumulative case counts data. Conceptually, the first-order difference accounts for the fact that case counts are accumulating, and the second-order difference accounts for the fact that cases accumulate at different rates during different points in the pandemic.

Next, we looked for aberrations in our data using a simple standard deviation-based outlier detection method. We fitted a normal distribution over the differenced data and flagged all instances of target data that were more than three standard deviations away from the mean of that distribution. We repeated this for all municipalities for the 11-day window centered on each of the three target dates that we used for the summary dataset. Of a possible 18,645 data points (565 municipalities × 3 target days × 11-day window per target) we had recorded values for 10,009 data points. Of these, we flagged 62 as possible errors. We manually examined each possible error. A total of 57 out of 62 data points had sparse data surrounding the target data; these therefore incorrectly triggered the outlier algorithm. The remaining 5 cases were deleted from the final data file. Only one of these data points fell on the target date selected by the algorithm. After removing this data point, we replaced it with the closest valid data point in the 11-day window.

We also note that the ``mean plus or minus three standard deviations'' method has been subjected to criticism [7], mainly on three fronts: (i) it assumed normality of data, (ii) it does not work for small sample sizes, and (iii) the mean and standard deviation are themselves affected by the outlier. While we broadly agree with these issues, the technique suffices for our purpose as (i) the differenced time-series data was reasonably approximated by a normal distribution, (ii) for most municipalities we have data over several days, and (iii) we only use this method to flag aberrantly large (or small) values due to error in data-collection and manually remove entries after evaluation.

## Ethics Statement

All data on COVID-19 cases are aggregated at the municipal level and were reported in public announcements.

## CRediT authorship contribution statement

**Yuqi Wang:** Conceptualization, Formal analysis, Visualization, Writing – original draft. **Sarah R. Allred:** Conceptualization, Formal analysis, Data curation, Visualization, Writing – review & editing, Supervision, Project administration, Funding acquisition. **Emily A. Greenfield:** Conceptualization, Writing – review & editing, Supervision, Funding acquisition. **Aayush Yadav:** Software, Formal analysis, Data curation. **Ryan Pletcher:** Investigation. **George Arthur:** Investigation. **Sachin Saxena:** Investigation. **Trista Harig:** Investigation. **Emily Rankin:** Investigation. **Benjamin Rudolph:** Investigation. **Ummulkhayer Sameha:** Investigation. **Shwetal Sharma:** Investigation. **Shibin Yan:** Investigation.

## Declaration of Competing Interest

The authors declare that they have no known competing financial interests or personal relationships which have or could be perceived to have influenced the work reported in this paper.

## Appendix Supplementary materials

Image, application 1

## References

[1] US Census Bureau, 2010 Decennial census. https://data.census.gov/cedsci/. Accessed May 1, 2021.

[2] S.R. Allred, et al., Research brief: municipal variation in COVID-19 case rates in New Jersey. https://rand.camden.rutgers.edu/files/COVID-research-muni-data_Brief-FINAL.pdf, 2021. Accessed September 1, 2021.

[3] Dong E., Du H., Gardner L. (2020). An interactive web-based dashboard to track COVID-19 in real time. Lancet Infect. Dis..

[4] Benitez J., Courtemanche C., Yelowitz A. (2020). Racial and ethnic disparities in Covid-19: evidence from six large cities. J. Econ. Race Policy.

[5] NJ Department of health, communicable disease reporting and surveillance system. https://www.nj.gov/health/cd/reporting/cdrss/, 2021. Accesssed May 1, 2021.

[6] Taylor S.J., Letham B. (2018). Forecasting at scale. Am. Stat..

[7] Leys C., Ley C., Klein O., Bernard P., Licata L. (2013). Detecting outliers: Do not use standard deviation around the mean, use absolute deviation around the median. J. Exp. Soc. Psychol..

