# Supplementary material for: New Jersey COVID-19 municipal dataset
Source: Data Brief. 2021 Sep 24;38:107426. doi: 10.1016/j.dib.2021.107426 (PMC8462753; doi:10.1016/j.dib.2021.107426)
Supplement: Supplementary file 1 [file mmc1.docx]

Data Sources for New Jersey Municipal COVID-19 Cases

Following is a complete list of all sources we used to collect municipal Covid-19 infections in NJ. The sources are arranged by counties. Some municipal data were reported in county websites, while some counties did not report municipal data and therefore we searched for alternative sources, primarily municipal websites. Frequency and regularity of data updates from the sources varied across counties and municipalities. We accessed data sources every one to three days to record available data for each municipality. Please note that some sources changed over time and some sources may be no longer reporting data.

| Atlantic County | Atlantic County website: <https://www.atlantic-county.org/> |
| --- | --- |
| Bergen County | Bergen County website: <https://www.co.bergen.nj.us/>  Bergen County Executive Facebook page: <https://www.facebook.com/JamesJTedesco/> |
| Burlington County | Burlington County COVID-19 Dashboard: <http://burlconj.maps.arcgis.com/apps/opsdashboard/index.html#/16e1e482d1a446a3b4f73fee4592b430> |
| Camden County | Camden County website: <https://www.camdencounty.com/service/covid-19-updates-and-preparations/most-recent-updates/> |
| Cape May County | Cape May County website: <https://capemaycountynj.gov/1388/COVID-19-Updates> |
| Cumberland County | Cumberland County COVID-19 dashboard: <https://cumberlandnj.maps.arcgis.com/apps/opsdashboard/> |
| Essex County | Essex County website: <https://essexcountynj.org/covid-19-municipality/> |
| Gloucester County | Gloucester County COVID-19 dashboard: <https://covid-19-glouc-cty-nj.hub.arcgis.com/?fbclid=IwAR347MGxJK5syZFAMmPf281hRxtMXHHNIdhnQbwrf1dOKvys6EFymChrJJ> |
| Hunterdon County | Hunterdon County website: <https://www.co.hunterdon.nj.us/> |
| Hudson County | The City of Bayonne website: <http://www.bayonnenj.org/>  Borough of East Newark website: <http://www.boroughofeastnewark.com/>  Town of Guttenberg website: <http://www.guttenbergnj.org/>  Town of Harrison website: <https://townofharrisonnj.com/AlertCenter.aspx>  The Hoboken Girl website: <https://www.hobokengirl.com/jersey-city-hoboken-coronavirus-updates-daily/>  Jersey City COVID-19 dashboard: <https://public.tableau.com/profile/jersey.city#!/vizhome/COVID-19CasesinJerseyCity/COVID-CasesinJerseyCity?publish=yes>  Town of Kearny website: <https://www.kearnynj.org/corona-virus-covid-19-updates/>  The Township of North Bergen website: <http://www.northbergen.org/>  The Town of Secaucus website: <https://www.secaucusnj.gov/virus#covid>  The City of Union City website: <http://www.ucnj.com/>  The Township of Weehawken website: <http://www.weehawken-nj.us/>  Town of West New York: <https://www.westnewyorknj.org/pages/covid-19> |
| Mercer County | Mercer County COVID-19 dashboard: <https://mercernj.maps.arcgis.com/apps/opsdashboard/index.html#/6a9746e0df254b7bb56083d827972321> |
| Middlesex County | Middlesex County website: <https://discovermiddlesex.com/total-cumulative-cases/> |
| Monmouth County | Monmouth County website: <https://www.co.monmouth.nj.us/page.aspx?ID=5012&mode=1> |
| Morris County | Morris County website: <https://www.morriscountynj.gov/Residents/Health/Coronavirus#cases> |
| Ocean County | Ocean County Health Department: <https://www.ochd.org/covid-19-case-totals/>  Ocean County COVID-19 Dashboard (old version): <https://insight.livestories.com/s/v2/covid-19-dashboard-ocean-county-nj/235d7065-93ef-441b-a62d-54c1813abd8a> |
| Passaic County | Passaic County website: <https://www.passaiccountynj.org/government/departments/health/current_health_alerts.php>  Passaic County Facebook page: <https://m.facebook.com/passaiccountynj/>  Clifton website: <https://www.cliftonnj.org/342/Coronavirus-2019-COVID-19>  Hawthorne website: <https://www.hawthornenj.org/485/COVID-19>  Township of Little Falls website: <https://www.lfnj.com/news-announcements/mayors-office/update-coronavirus-may-3-2021-little-falls-covid-19-update>  Borough of North Haledon website: <https://www.northhaledon.com/alert_detail.php>  City of Passaic website (no longer available): <https://www.cityofpassaic.com/index.asp?SEC=C5BE05AF-3DB9-4841-9151-307A6C48CBA4&DE=B883FB8F-E4C3-421B-933B-AE3B163622B8>  Borough of Ringwood website: <http://www.ringwoodnj.net/content/2347/2357/18377.aspx>  Borough of Totowa: <http://totowanj.org/CoronaVirus.html>  Township of Wayne: <https://www.waynetownship.com/26-homepage/488-corona-virus-update.html> |
| Salem County | Salem County Department of Health and Human Services Facebook page: <https://www.facebook.com/SalemCountyHealth/> |
| Sommerset County | Somerset County website: <https://somerset-county-nj-coronavirus-response-somerset.hub.arcgis.com/>  TAPintoSomerville news: <https://www.tapinto.net/towns/somerville/sections/health-and-wellness/articles/sept-10-somerset-county-covid-19-update-13-new-cases-overnight>  My central jersey news: <https://www.mycentraljersey.com/story/news/health/2020/09/15/nj-covid-19-somerset-county-town-town-cases-deaths-sept-15/5802690002/> |
| Sussex County | Sussex County website: <https://sussex.nj.us/cn/webpage.cfm?tpid=17089&utm_source=covid19&utm_medium=web&utm_campaign=coronavirus>  Insider NJ news: <https://www.insidernj.com/sussex-county-reports-760-covid-19-cases-total-wednesday/> |
| Union County | Berkeley Height website: <https://berkeleyheights.gov/1429/COVID-19-Statistics>  Clark Township website (no longer available): <https://www.ourclark.com/civicalerts.aspx?aid=88>  Cranford website: <https://www.cranfordnj.org/covid-19-information-resources/pages/covid-19-updates>  Elizabeth website: <https://www.elizabethnj.org/CivicAlerts.aspx?AID=41>  Fanwood website: <https://www.fanwoodnj.org/covid-19/>  Garwood Borough Government Facebook page: <https://www.facebook.com/garwoodboroughgovernment/>  Councilman Cook (of Hillside) Facebook page: <https://www.facebook.com/gcook1>  The City of Linden website: <https://linden-nj.gov/covid-19-confirmed-case-data/>  Mountainside TV YouTube channel: <https://www.youtube.com/user/MountainsideTV/videos>  Borough of New Providence website: <https://www.newprov.org/case-statistics/>  City of Plainfield website: <https://plainfieldnj.gov/cms.aspx?page_id=435>  Rahway dashboard: <https://www.arcgis.com/apps/dashboards/dd79310c6c934e5c995d8396be25e219>  Borough of Roselle website: <https://www.boroughofroselle.com/_Content/pdf/Roselle-COVID19-Cases-as-of-03-02-2021.pdf>  Roselle Park website: <https://www.rosellepark.net/CORONAVIRUS/>  Township of Scotch Plains website: <https://www.scotchplainsnj.gov/coronavirus/>  Township of Springfield website: <https://springfield-nj.us/latest-news-covid-19/>  City of Summit website: <https://www.cityofsummit.org/660/COVID-19>  UNION TV34: <https://vimeo.com/uniontv34>  Town of Westfield website: <https://www.westfieldnj.gov/coronavirusupdates> |
| Warren County | Warren County website: <http://www.co.warren.nj.us/> |
